# The apoptosis associated tyrosine kinase gene is frequently hypermethylated in human cancer and is regulated by epigenetic mechanisms

**DOI:** 10.18632/genesandcancer.28

**Published:** 2014-09

**Authors:** Tanja Haag, Christina E. Herkt, Sara K. Walesch, Antje M. Richter, Reinhard H. Dammann

**Affiliations:** ^1^ Institute for Genetics; Justus-Liebig-University; Universities of Giessen and Marburg Lung Center, Member of the German Center for Lung Research; Giessen, Germany

**Keywords:** AATK, epigenetic regulation, DNA methylation, human cancer, tumor suppressor, CTCF

## Abstract

Epigenetic gene inactivation through promoter hypermethylation is an important aberration involved in the silencing of tumor-associated genes in cancer. Here we identified the *apoptosis associated tyrosine kinase (AATK)* as an epigenetically downregulated tumor related gene. We analyzed the epigenetic regulation of *AATK* in several human cancer cell lines and normal tissues by methylation and expression analysis. Hypermethylation of *AATK* was also analyzed in 25 primary lung tumors, 30 breast cancers and 24 matching breast tissues. In normal tissues the *AATK* CpG island promoter was unmethylated and *AATK* was expressed. Hypermethylation of *AATK* occurred frequently in 13 out of 14 (93%) human cancer cell lines. Methylation was reversed by 5-aza-2′-deoxycytidine treatment leading to re-expression of *AATK* in cancer cell lines. Aberrant methylation of *AATK* was also revealed in primary lung (40%) and breast (53%) cancers, but was found to be significantly less methylated in matching normal breast tissues (17%; p<0.01). In addition, we observed that *AATK* is epigenetically reactivated through the chromatin regulator CTCF. We further show that overexpression of Aatk significantly suppresses colony formation in cancer cell lines. Our findings suggest that the *apoptosis associated tyrosine kinase* is frequently inactivated in human cancers and acts as a tumor suppressive gene.

## INTRODUCTION

Epigenetic modifications are important regulatory mechanisms for initiating and maintaining memory effects on gene expression. In mammals, these epigenetic mechanisms play essential roles in normal development through their effect on gene imprinting, X-chromosome inactivation and transcriptional inactivation of repetitive genomic elements. Moreover, epigenetic silencing of tumor suppressor genes is frequently observed in cancer [1]. In particular, *de novo* methylation of CpG island promoters is a hallmark of gene silencing during malignant transformation. CpG islands are sequences greater than 500 bp of GC-rich and CpG-dense elements in the genome. About 70% of known genes harbor a CpG island within −1 kb to +1 kb of their transcription initiation site. During tumorigenesis CpG island promoters become hypermethylated and this alteration is accompanied by the formation of a repressive chromatin and transcriptional silencing. Tumor suppressor genes that are frequently epigenetically inactivated are the *Ras association domain family 1A* (*RASSF1A*) gene and the *cyclin-dependent kinase inhibitor 2A* (p16) [1-3].

The CCCTC binding factor (CTCF) is a zinc finger-encoding protein involved in imprinting and chromosomal gene organization [4]. Several findings suggest that the CTCF insulator protein may contribute the boundaries at CpG island promoters [5-8]. Binding of CTCF is maintained in mitotic chromatin and may provide an epigenetic memory during cell division of proliferating cells [9]. Disruption of molecular boundaries mediated by CTCF may facilitate the epigenetic silencing of tumor suppressor genes [10, 11]. Recently, it has been shown that epigenetic downregulation of *p16* and *RASSF1A* is associated with loss of CTCF binding and disappearance of a chromatin boundary [12].

**Fig. 1:**
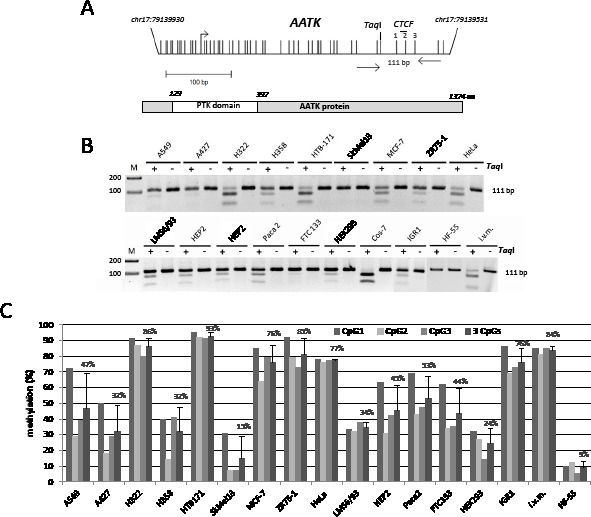
Hypermethylation of AATK in human cancers A. Structure of the *AATK* CpG island promoter on chromosome 17 and the AATK protein. Arrows mark transcriptional (+1) start site for *AATK.* Vertical lines indicate CpGs. The 111 bp PCR product with respective primers and the *Taq*I site are depicted. The CTCF binding site is shown. The protein tyrosine kinase (PTK domain) of AATK is marked. B. Combined bisulfite restriction analysis of *AATK*. Bisulfite-treated DNA from the indicated cancer cell lines, human fibroblasts (HF-55) and *in vitro* methylated DNA (i.v.m.) was amplified, digested with *Taq*I (+) or mock digested (−) and resolved on 2% gels with a 100 bp marker (M). C. Bisulfite pyrosequence analysis of *AATK*. The methylation levels of three CpGs of the PCR products were analyzed by pyrosequencing.

The *apoptosis associated tyrosine kinase* (*AATK*) gene is localized on chromosome 17 at q25.3 [13]. Deletion of 17q25.3 was reported for several human cancers including oral, cervical and breast cancer [14-16]. The AATK protein that is also named AATYK or LMTK1 (lemur tyrosine kinase 1) consists of 1374 aa with the N-terminus harboring a protein tyrosine kinase domain (Fig. 1) [17]. AATK promotes neuronal differentiation and is induced during growth arrest and apoptosis of myeloid cells [18, 19]. Downregulation of *AATK* expression was reported for adenocarcinoma of the colon and for melanomas [20, 21]. In melanoma cells AATK overexpression inhibits growth and migration, and promotes apoptosis [21]. In our study we report frequent epigenetic inactivation of *AATK* in different human cancer entities (e.g. breast and lung) and its growth suppressive function in lung cancer. Furthermore, we show that the chromatin regulator CTCF induces epigenetic reactivation of *AATK*.

## RESULTS

### Methylation of AATK occurs in human cancers

We have performed a genome wide methylation screen in the lung cancer cell line H322 and have revealed a promoter specific hypermethylation of the *apoptosis associated tyrosine kinase* (*AATK*) gene (data not shown). The schematic promoter region of *AATK* and corresponding CpG islands is shown in Fig. 1A. The promoter lies within a CpG island of 527 bp on chromosome 17 from position 79′139′502 to 79′140′028 (UCSC genome browser).

To reveal the epigenetic status of *AATK* in human cancers in more detail, we have analyzed the aberrant methylation of *AATK* in lung cancer (A549, A427, H322, H358, HTB-171), breast cancer (MCF-7, ZR75-1), melanoma (Sk-Mel13, IGR1), leiomyosarcoma (LMS6/93), follicular thyroid (FTC133), larynx cancer (HEP2), pancreas carcinoma cell line (PaCa2), cervix cancer (HeLa), HEK293 and human fibroblast (HF-55) by COBRA (Fig. 1B). Fragmentation of the PCR product indicates an underlying methylated *AATK*, whereas undigested PCR products originate from unmethylated *AATK* CpG island. *In vitro* methylated genomic DNA (i.v.m.) served as a methylated control (Fig. 1B). Normal human fibroblast (HF-55) and melanoma cells (Sk-Mel13) were unmethylated as analyzed by COBRA. COBRA data were confirmed by genomic bisulfite pyrosequencing of three CpGs within the *AATK* CpG island promoter (Fig. 1A and C). All lung cancer cell lines (A549, A427, H322, H358 and HTB171) were partially methylated for *AATK* (Fig. 1B and C). For breast cancer cell lines (MCF-7, ZR75-1), HeLa, LMS6/93, HEP2, Paca2, FTC133 and IGR1 partial methylation of *AATK* was also observed (Fig. 1B and C). Thus, a total of 15 cancer cell lines were analyzed, of which 14 (93%) were methylated for *AATK*. Therefore frequent hypermethylation of *AATK* was found in different human cancer entities, including lung and breast cancers.

### AATK hypermethylation in primary human breast and lung cancers

**Fig. 2:**
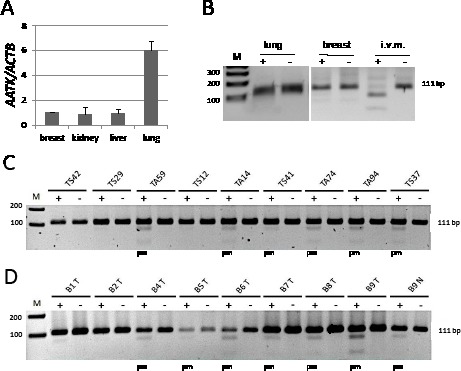
Expression and methylation of AATK in normal tissues and primary tumors A. Expression of *AATK* was analyzed in normal breast, kidney, liver and lung tissues by quantitative RT-PCR and normalized to *ACTB* levels (breast=1). B. Methylation of *AATK* was analyzed in normal lung and breast tissues and *in vitro* methylated DNA by COBRA. Mock digest (−) and *Taq*I digest (+) are shown. C. Methylation analysis is shown for primary lung cancer samples (TA=adenocarcinoma, TS=squamous cell carcinoma). D. Methylation analysis in primary breast cancer (T=tumor, N=corresponding normal tissue). Products were resolved on a 2% gel with a 100 bp marker (M). (pm=partially methylated).

To analyze the impact of epigenetic regulation of *AATK* in carcinogenesis we investigated its expression and methylation in normal tissues as well as in breast and lung cancer samples (Fig. 2). Expression of *AATK* was found in normal breast, kidney and liver tissues and the highest expression was observed in normal lung tissues (Fig. 2A). *AATK* was unmethylated in normal lung and in three breast tissues isolated from healthy patients (Fig. 2B and data not show). Next we analyzed the aberrant methylation of *AATK* in 25 primary lung and 30 breast cancers by COBRA (Fig. 2). Four out of five lung adenocarcinomas (e.g. TA59) exhibited a partial methylation of *AATK* (Fig. 2C). However, aberrant *AATK* methylation was found in only two out of eight squamous lung tumors (e.g. TS37; Fig. 2C). In small cell lung cancer *AATK* was hypermethylated in four out of 12 tissues (data not shown). Thus, aberrant methylation of *AATK* was observed in 10 out of 25 (40%) of lung cancer samples. We also analyzed 30 primary breast tumors and 24 corresponding matching tissue controls (Figure 2D). 16 out of 30 (53%) breast cancer tissues were methylated, but *AATK* methylation was only found in 4 out of 24 (17%) matching control tissues (p<0.01, two tailed Fisher's exact test). Moreover, methylation was weaker in the normal samples than in the corresponding tumor tissue (e.g. B9N and B9T, respectively; Fig. 2D). In summary, hypermethylation of *AATK* was also demonstrated in primary tumor tissues of cancer patients.

### Decreased expression of AATK is associated with its hypermethylation in human cancer cell lines

**Fig. 3:**
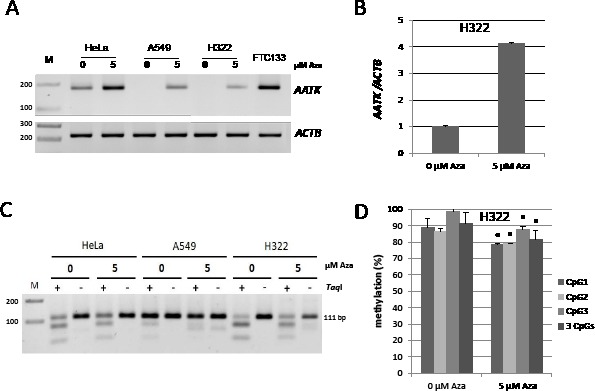
AATK expression in cancer cells, reexpression and demethylation under Aza treatment A. Expression analysis of *AATK* is shown after Aza treatment (0 and 5 μM) in HeLa, lung cancer cell lines A549 and H322 and thyroid cancer cell line FTC133. *AATK* (170 bp) and *ACTB* (226 bp) levels were analyzed by RT-PCR after four days of Aza treatment on a 2% gel. B. Expression of *AATK* was analyzed in H322 cells by quantitative RT-PCR after Aza treatment and normalized to *ACTB*. C. COBRA analysis is shown after Aza treatment. Product of *Taq*I digest (+) and mock digest (−) were resolved on a 2% gel with a 100 bp marker (M). D. *AATK* methylation analysis was performed in lung cancer H322 and quantified by bisulfite pyrosequencing. Three CpGs are included in analyzed region and according mean and SD are shown. P values were calculated using two tailed t-test (* = p<0.05).

5-Aza-2′-deoxycytidine (Aza) inhibits DNA methylation [22] and is known to reverse hypermethylation of tumor suppressor genes, which can further lead to their re-expression [2]. We therefore chose HeLa, A549 and H322 with a methylated *AATK* CpG island for Aza treatment and analyzed *AATK* expression by RT-PCR as well as its methylation status (Fig. 3). The lung cancer cell line H322 has a methylated *AATK* promoter (Fig. 1 and 3) and shows very low endogenous *AATK* expression (Fig. 3A and B). RT-PCR shows that 5 μM Aza leads to *AATK* re-expression (Fig. 3A and B). Similar results are observed for the lung cancer cell line A459 and for HeLa cells, which are partially and strongly methylated respectively (Fig. 1 and 3). In HeLa and A549 the *AATK* expression is increased upon Aza treatment (Fig. 3A). FTC133 is partially methylated for the *AATK* promoter (Fig. 1B) and *AATK* expression is comparable to the Aza-treated HeLa cells (Fig. 3A). The re-expression under Aza treatment for HeLa, A549 and H322 was accompanied by *AATK* demethylation (Fig. 3C and D). H322 were 92% methylated and under Aza treatment methylation decreased significantly to 82% (5 μM Aza) (p=0.01, t-test; Fig. 3D). Genomic bisulfite pyrosequencing revealed a significant 10% demethylation at all three analyzed CpGs (p<0.05; Fig. 3D). In cancer cells, we observed an epigenetic silencing of the *AATK* CpG island promoter, which was reversed by Aza treatment as shown by AATK re-expression and promoter demethylation.

### Regulation of AATK by the insulator protein CTCF

**Fig. 4:**
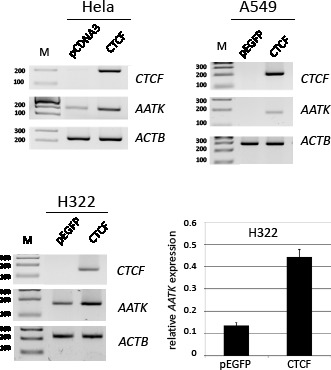
CTCF induced reexpression of AATK Expression analysis of HeLa, lung cancer cells A549 and H322 after CTCF and control transfection. CTCF was cloned in the corresponding vector (pCDNA3 or pEGFP) and transfected. After one to two days RNA was isolated and expression of *AATK* (170 bp), *CTCF* (199 bp) and *ACTB* (226 bp) was analyzed by RT-PCR on a 2% gel with a 100 bp ladder (M). For H322 *AATK* levels were analyzed by qRT-PCR and normalized and plotted relative to *ACTB* (=1).

**Fig. 5:**
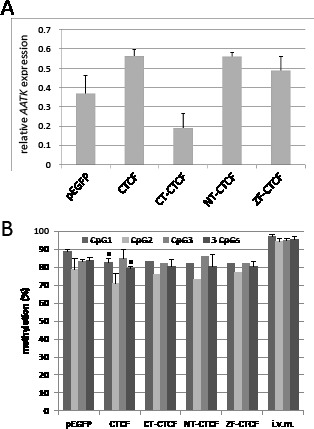
CTCF induced reexpression and demethylation of AATK A. Expression analysis of *AATK* after CTCF and control transfection. Different constructs of chicken CTCF (CT=C-terminal domain, NT=N-terminal domain and ZF=zinc finger domain) were generated and transfected in H322 lung cancer cells. After two days RNA was isolated and *AATK* expression was analyzed by qRT-PCR and then normalized and plotted relative to *ACTB* (=1) B. CTCF induced demethylation of *AATK*. Methylation analysis was performed in H322 after CTCF and control (pEGFP) transfection. The average methylation of three CpGs was quantified by bisulfite pyrosequencing. P values were calculated using two tailed t-test. (i.v.m. = in vitro methylated DNA control; * = p<0.05).

Previously, it has been reported that the CTCCC binding protein (CTCF) is involved in the epigenetic regulation of tumor suppressor genes [12]. Wendt and Barksi et al. have reported that downregulation of CTCF by RNA interference caused a twofold repression of *AATK* in HeLa cells [23, 24]. Database analysis of CTCF ChipSeq Encode data at the CTCFBSDB2.0 site revealed that CTCF binds the *AATK* CpG island in HUVEC. Furthermore, a search for potential CTCF-binding sites at the *AATK* locus found a match for a CTCF binding consensus site from position 79′139′590 to 79′139′599 (CCGCCAGGG) at CpG site 2 (Fig. 1A) (http://bsproteomics.essex.ac.uk: 8080/bioinformatics/ctcfbind.htm). To clarify whether CTCF could regulate *AATK* expression, we transfected CTCF in HeLa, A549 and H322 cancer cells (Fig. 4). Expression of CTCF induced the expression of *AATK* considerably in all three cell lines (Fig. 4). For H322 cells a 3.3 time increase in AATK expression was observed (Fig. 4). Since CTCF consist of three distinct domains: an N-terminal domain (NT), a zinc finger domain (ZF), which binds to DNA and a C-terminal (CT) domain. We generated deletion constructs of CTCF to investigate which of these domains is responsible for reactivation of *AATK*. Subsequently, we transfected these deletion constructs (CT-, NT- and ZF-CTCF), full length CTCF, and the empty EGFP vector control in H322 lung cancer cells (Fig. 5). Transfection of CTCF, NT-CTCF and ZF-CTCF induced the expression of *AATK* to levels similar to that of the control (Fig. 5A). Interestingly, a repression of *AATK* expression occurred for CT-CTCF transfection. To reveal if this induction is accompanied by changes in methylation levels we performed bisulfite pyrosequencing of three CpG sites (CpG1, CpG2 and CpG3) around the CTCF consensus site (Fig. 1A and Fig. 5B). Interestingly, we observed a slight and significant decrease from 84% to 79% average methylation of the three CpGs after control and CTCF transfection, respectively (p=0.03, t-test; Fig. 5B). This decrease was higher for CpG1 (89% to 83%; p=0.03) and CpG2 (79% to 71%, p=0.3), than for the third CpG (Fig. 5B). Similar demethylation was also observed after CT-CTCF, NT-CTCF and ZF-CTCF transfection (Fig. 5B). It is interesting to note that demethylation of the CpG site 2, which harbors the CTCF consensus site, was more pronounced after CTCF and NT-CTCF transfection compared to CT-CTCF and ZF-CTCF. In summary, we observed a reproducible demethylation of *AATK* after CTCF overexpression.

### AATK expression reduces colony formation of cancer cells

**Fig. 6:**
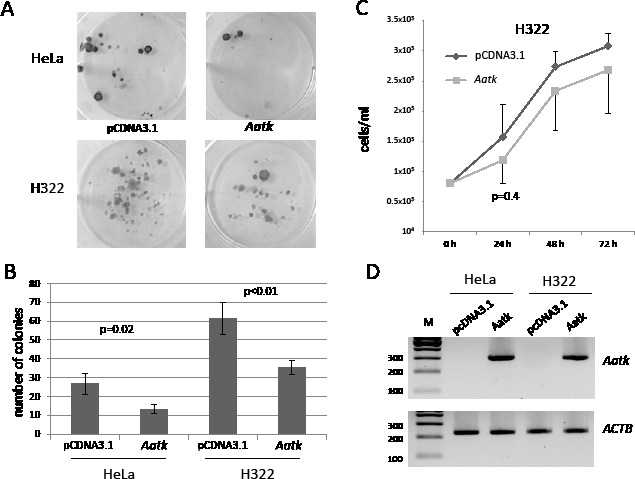
Overexpression of Aatk reduces colony formation and proliferation of cancer cell lines A. Colony formation after Aatk reexpression in cancer cells. HeLa and H322 lung cancer cell lines were transfected with *Aatk* or empty vector (pCDNA3.1), selected with G418 for 28 days and Giemsa stained. B. Colony formation experiment was repeated three times, and the mean colony numbers with according SD are shown for HeLa and H322 cells. P values were calculated using two tailed t-test. C. Proliferation of H322 cells after transient Aatk transfection. 0.8 × 10^5^ cells/ml were transfected with *Aatk* or empty vector (pCDNA3.1) and cells were counted for three days in a hemocytometer. Experiments were performed in triplicate and the corresponding mean and SD are plotted. D. Overexpression of *Aatk* was confirmed by RT-PCR in HeLa and H322 cells. HeLa and H322 cells were transfected with the indicated constructs and RNA was isolated. *Aatk* (290 bp) and *ACTB* (226 bp) levels were analyzed on a 2% gel together with a 100 bp marker (M).

To functionally test AATK and its ability to suppress tumor formation, we performed colony formation and proliferation assays (Fig. 6). In order to do so we transfected HeLa and H322 with the *Aatk* expression- or empty control construct (pCDNA3.1) and selected with G418 for three weeks. Colonies were Giemsa stained and representative pictures are shown (Fig. 6A). In both cell lines *AATK* is methylated and downregulated (Fig. 1 and 3). After transfection of the Aatk containing construct, its expression was detected on RNA level (Fig. 6D). Expression of *Aatk* in these two cell lines significantly reduces the number of colonies (Fig. 6B). For both cell lines a twofold reduction in colonies was found (Fig. 6B). Moreover we analyzed proliferation of H322 cells after transient Aatk transfection. Cells were transfected with *Aatk* or empty vector (pCDNA3.1) and were counted over three days (Fig. 6D). After 24 hours a 25% reduction in cell number was found, however this reduction was not significant due to the observed variation (p=0.4). The significant reduction of colonies suggests that Aatk exhibits a tumor suppressive function in cancer cells.

## DISCUSSION

In our study, we have identified the *apoptosis associated tyrosine kinase* (*AATK*) gene as a novel epigenetically inactivated target gene in human cancer. Hypermethylation of *AATK* was found in several epithelial cancer entities including lung, breast, skin, cervix, larynx and pancreatic cancer (Fig. 1 and Fig. 2). However, expression of *AATK* was found in normal tissues and its CpG island was unmethylated (Fig. 2). We observed that aberrant methylation of *AATK* is also frequently found in primary lung cancer (40%) and breast cancers (53%). Methylation frequency was significantly lower in matching normal tissues compared to breast cancers (17% compared to 53%, p<0.01). It is interesting to note that deletion of the chromosomal region which harbors the *AATK* gene is observed in different tumor entities including breast, cervix and oral cancers [14-16]. Since in general both alleles of a tumor suppressor gene are inactivated, these findings indicate that deletion and epigenetic inactivation are two important pathways for the inactivation of *AATK* in human cancer. It will be interesting to analyze if single nucleotide substitutions are present in the coding or regulatory sequences of *AATK*. Previously, downregulation of AATK at the protein level was only observed in colon polyps and melanoma, however the mechanism responsible for this downregulation was not revealed in detail [20, 21]. Thus, *AATK* hypermethylation may also be present in colon cancer and melanoma, as observed for the *RASSF1A* gene and other tumor suppressor genes [25-27]. Methylation of *AATK* has been reported in low grade serous ovarian neoplasms [28]. Here we report that *AATK* hypermethylation is frequently found in other human epithelial cancers, including primary breast and lung cancers (Fig. 2). Thus, it will be interesting to evaluate whether aberrant *AATK* methylation may represent a novel biomarker for prognostic or diagnostic purposes in human cancer.

By using 5-aza-2′-deoxycytidine treatment we were able to demethylate the *AATK* CpG island promoter and to re-express *AATK* (Fig. 3). This suggests that downregulation of *AATK* in cancer cell lines is due to its promoter hypermethylation. Moreover, we observed that expression of the epigenetic regulator CTCF also induced the expression and demethylation of *AATK* (Fig. 4 and Fig. 5). Previously, it has been reported that knockdown of *CTCF* by RNA interference caused twofold repression of *AATK* in HeLa cells [23, 24]. Here we show that overexpression of CTCF induced the expression of *AATK* in HeLa and in the lung cancer cell lines A549 and H322. It has been postulated that CTCF acts as a tumor suppressive factor [29, 30], since its function has been associated with altered expression of tumor-suppressor gene, such as *E-cadherin, retinoblastostoma, RASSF1A* or *p16/CDKN2A* [10, 12]. Disrupting the spectrum of target specificities of CTCF by mutations, its aberrant modifications (e.g. PARylation) or abnormal selective methylation of targets (e.g. loss of imprinting) could be associated with cancer [12, 31, 32]. Recurrent mutations of CTCF are mostly clustered in the conserved zinc finger domain [30]. It has been reported that CTCF-defective PARylation and dissociation from the molecular chaperone Nucleolin occur in p16-silenced cells, abrogating its function [12]. Using CTCF mutants, the requirement of PARylation for optimal CTCF function has been demonstrated in transcriptional activation of the *p19ARF* promoter and inhibition of cell proliferation [33]. In our experiments we observed that full length CTCF and the N-terminal domain of CTCF, which harbor a intact PARylation site, induce the expression of *AATK*. In contrast, the C-terminal site of CTCF repressed *AATK* expression. CTCF has also been reported to act as a gene silencer [34]. Thus, the function of CTCF may require a specific composition of factors (e.g. PARP1) at CTCF binding sites. This has been suggested in a model where PARylation of CTCF is involved in gene activation [12]. Others have reported that SUMOylation of CTCF modulates a domain in CTCF that activates transcription and decondenses chromatin [35]. One SUMOylation site is also found in the N-terminal domain of CTCF. Thus, CTCF may act as a tumor relevant factor by inducing tumor relevant genes such as *AATK*. In the case of *AATK* this induction was also accompanied by a significant demethylation (Fig. 5B). Chipseq data reveal a CTCF binding site within the analyzed sequence. Thus, it will be interesting to analyze if increased CTCF binding occurs at its binding site upon CTCF expression. It has been shown that CTCF bound DNA remains unmethylated [36]. Stadler et al. showed that CTCF can bind to a pre-methylated CpG-poor target site, which in turn leads to localized demethylation [36]. However the exact mechanism of this local demethylation event has not been elucidated, and it could be passive by inhibiting the accessibility of binding sites for DNA methyltransferases during DNA replication. For this aspect, it is important to note that the demethylation caused by CTCF (5%) was half as much compared to Aza (10%), however Aza treatment took twice as long (4 days) compared to CTCF transfection (2 days). Additional chromatin changes (e.g. histone modification or structural alteration) could be involved in the CTCF-induced expression of *AATK* and should to be analyzed in further studies.

To verify the ability of AATK to suppress tumor growth as do other tumor suppressor genes, we performed colony formation and proliferation assays in human cancer cell lines (Fig. 6). Our results show that Aatk significantly suppresses colony growth in H322 lung cancer cells and HeLa cells, in which *AATK* is downregulated and inactivated by aberrant promoter methylation (Fig. 1 and Fig. 2). Recently, it has been reported that AATK inhibits cell proliferation, colony formation, migration, and also promotes apoptosis in melanoma cells [21]. Since we observed hypermethylation of AATK in IGR1 melanoma cell lines (Fig. 1), it will be interesting to analyze its methylation status in primary melanomas.

AATK was characterized as a novel kinase that induces and promotes neuronal differentiation in a human neuroblastoma cell line [19]. Thus it will be important to analyze if downregulation of AATK is associated with dedifferentiation of human epithelial cells. It has been reported that AATK interacts with the p35 activation subunit of the cyclin-dependent kinase 5 (CDK5) and is then phosphorylated by CDK5 [37, 38]. It has been suggested that AATK is a regulator of axonal outgrowth involving the RAB11 endosomal recycling pathway [17]. However, it is not known whether AATK phosphorylates any target proteins, and the function of AATK in epithelial cells has not been analyzed in detail.

In our study we demonstrate that *AATK* is frequently hypermethylated in human cancer cell lines and in primary lung and breast cancer samples. Demethylation of *AATK* is accompanied by re-expression of *AATK* in cancer cell lines. *AATK* expression was further found to be epigenetically regulated by CTCF. Additionally, ectopic expression of AATK suppresses colony formation. It will be fascinating to investigate the functional relationship of *AATK* epigenetic inactivation in cancer cell lines and their inability to differentiate or to undergo apoptosis. Therefore the loss of *AATK* might even promote dedifferentiation during tumorigenesis. Future research will elucidate the function of AATK during carcinogenesis.

## MATERIALS AND METHODS

### Tissue and cell lines

Primary cancer tissues and cancer cell lines were previously published: breast and lung [39-42]. All patients signed informed consent at initial clinical investigation. The study was approved by local ethic committees (City of Hope Medical Center, Duarte, USA and Martin-Luther University, Halle, Germany). All cell lines were cultured in a humidified atmosphere (37°C) with 5% CO_2_ and 1×Penicillin/Streptomycin in the recommended medium. Cells were transfected with 4 μg or 10 μg of constructs for 3.5 or 10 cm plates, respectively using Polyethylenimine or Turbofect (Fermentas GmbH, St.Leon-Rot, Germany).

### Methylation analysis

DNA was isolated by phenol-chloroform extraction and then bisulfite treated prior to COBRA analysis and pyrosequencing [43]. 200 ng were subsequently used for PCR with primer AATKBSU1 (GGTTTGTATGGAAATTAATTTTTTTTT) and 5′-biotinylated primer AATKBSL2 (ATTTATACTAAAACCCAAAACCTACCC). Products were digested with 0.5 μl *Taq*I (Fermentas GmbH, St.Leon-Rot, Germany) 1 h at 65°C and resolved on 2% TBE gel. Methylation status was quantified utilizing the primer AATKSeq1 (GAGTTTAGTAGTAGAAGTAGT) and PyroMark Q24 (Qiagen, Hilden, Germany). Three CpGs are included in analyzed region of *AATK* and mean methylation was calculated. For *in vitro* methylation of genomic DNA we used M.SssI methylase (NEB, Frankfurt, Germany).

### Expression analysis

RNA was isolated using the Isol-RNA lysis procedure (5 Prime, Hamburg, Germany). 25 μg of breast, kidney, liver and lung RNA of normal human samples (pools of five, one, three and four, respectively) were obtained from Agilent Technologies (Waldbronn, Germany). RNA was DNase (Fermentas GmbH, St.Leon-Rot, Germany) digested and then reversely transcribed [44]. RT-PCR was performed with primers: AATKRTF1: TGGCCTGGCTCACTGCAAGTACAG, AATKRTR1: CCCAGATGGTCACGCCCAGG, mAatkRTF1: GTGCTGAAGTGACCCCCTAC, mAatkRTR1: GGTCAGCGGTCACGAGATAG, ßACTFW: CCTTCCTTCCTGGGCATGGAGTC, ßACTRW: CGGAGTACTTGCGCTCAGGAGGA, GGCTCFRTFW: CAGGAAACGGAGGCTACGGTGG, GGCTCFRTRW: CCTCCTGCAGGCCTCCTTTGGA. Quantitative PCR (qRT-PCR) was performed in triplicate with PerfeCTa SYBR® Green (Quanta BioSciences, Gaithersburg, USA) using a Rotor-Gene 3000 (Corbett Research, Qiagen, Hilden, Germany).

### Constructs

The cDNA of *Aatk* was obtained as a full length cDNA vector IRAVp968F06121D (Accession Number: BC080846; 5277 bp in pYX-Asc; imaGenes GmbH, Berlin, Germany) and cloned into the NotI and EcoRI sites of pcDNA3.1+. CTCF was a generous gift from Rainer Renkawitz (Justus Liebig University, Giessen, Germany) and deletion mutations were generated with QuickChange Lightning Site-Directed Mutagenesis Kit (Promega, Heidelberg, Germany).

## List of Abbreviations

AATK: apoptosis associated tyrosine kinase
COBRA: combined bisulfite restriction analysis
Aza: 5-aza-2′-deoxycytidine
CTCF: CCCTC binding factor
